# Public Preferences for Exit Strategies From COVID-19 Lockdown in Germany—A Discrete Choice Experiment

**DOI:** 10.3389/ijph.2021.591027

**Published:** 2021-03-19

**Authors:** Christian Krauth, Carina Oedingen, Tim Bartling, Maren Dreier, Anke Spura, Freia de Bock, Ursula von Rüden, Cornelia Betsch, Lars Korn, Bernt-Peter Robra

**Affiliations:** ^1^ Institute for Epidemiology, Social Medicine and Health Systems Research, Hannover Medical School, Hannover, Germany; ^2^ Center for Health Economics Research Hannover (CHERH), Hannover, Germany; ^3^ Federal Centre for Health Education (BZgA), Cologne, Germany; ^4^ Heisenberg-Professorship of Health Communication, University of Erfurt, Erfurt, Germany; ^5^ Institute of Social Medicine and Health Systems Research, Otto-von-Guericke-University of Magdeburg, Magdeburg, Germany

**Keywords:** COVID-19, exit strategies, discrete choice experiment, preferences, Germany

## Abstract

**Objectives:** To decrease the rapid growth of SARS-CoV-2 in Germany, a stepped lockdown was conducted. Acceptance and compliance regarding entering and exiting lockdown measures are key for their success. The aim of the present study was to analyse the population's preferences for exiting lockdown measures.

**Methods:** To evaluate population’s preferences and identify trade-offs between different exit strategies, a discrete choice experiment was conducted on 28–29 April (n = 1,020). Overall, six attributes and 16 choice sets (fractional-factorial design) without an opt-out were chosen. Conditional logit and latent class models were conducted.

**Results:** Most attributes proved to be significant. Two attributes dominated all others: Avoiding a mandatory tracing app, and providing sufficient intensive care capacities. Preventing a high long-term unemployment rate and avoiding the isolation of persons aged 70+, were relevant, though utilities were comparatively lower. We identified subgroups (elderly persons and persons with school children) with different utilities, which indicates specific attributes affecting them dissimilarly.

**Conclusions:** The population prefers cautious re-opening strategies and is at least sceptical about the adoption of severe protection measures. Government should balance interests between subgroups.

## Introduction

SARS-CoV-2 is a worldwide pandemic affecting Europe since end of January 2020. As many other European countries, Germany was confronted with exponential growth of infection and COVID-19 death numbers during the first few weeks [1]. Thus, as most countries in Europe, Germany has closed public areas. However, this was less restrictive than countries which reported higher case numbers and overloaded hospital intensive care units (ICU), such as Italy, France and Spain [2, 3].

Between March, 9 and 23 a stepped lockdown was conducted, starting with the prohibition of mass meetings (e.g. sports events, fairs and congresses). In the next steps, public areas such as schooling, retail sector (excepting food retailers) and gastronomy were closed, most activities prohibited, travelling restrained to a minimum, and borders to the neighbouring countries closed. Enterprises reduced their production due to diminishing demand and sent their employees to work from home. Finally, strict contact limitations and social distancing (of 1.5–2 m) were decreed. Furthermore, to prevent an overload of the hospital sector, elective surgeries were postponed and additional ICU capacities generated.

New daily cases peaked March, 16, while the number of deaths peaked April, 7 and declined steadily afterwards [1]. An overload of ICU capacities had been avoided [4, 5]. Exit strategies intended to ease re-opening of public areas were discussed in the broader public or were already announced by the government, as targeting economic (e.g., avoiding a long-term recession) and healthcare goals (providing usual healthcare to non-COVID-19 diseases and reducing psychosocial problems due to lockdown measures) regained importance [6, 7]. From April, 15 additional protection measures (like mandatory face masks or a tracing app) were discussed in the public as well as in government resulting in an obligation to use face masks in public areas by April, 27.

The measures were accompanied by a broad public discourse [8]. Population’s acceptance of and compliance with measures are key factors for the success of governmental strategies entering and exiting a lockdown [9]. For governments, it is important to understand population’s preferences and gain their trust in governmental strategies [10]. Therefore, the weekly COVID-19 Snapshot Monitoring (COSMO) provided the German government and other relevant stakeholders with timely data on acceptance of measures, trust, risk perceptions, worries, and other relevant aspects of managing the COVID-19 situation [11]. The aim of the present study is to analyse the population’s preferences for exit lockdown measures. We present these preferences as of April, 28–29, applying a discrete choice experiment (DCE) as part of the COSMO survey [12].

## Methods

The DCE is a stated-preference method often used in health economics research to elicit preferences for healthcare services and products [13]. It is an attribute-based-method asking respondents about different scenarios, designed to mimic real life and to make hypothetical, but realistic choices on the basis of their own preferences between different alternatives in each scenario. DCEs are based on theory of consumer choice [14] which assumes that [a] the utility of goods and services can be described or defined by different key characteristics or factors (i.e., attributes) that characterise the good or service and that [b] each attribute varied systematically with different specifications (i.e., attribute levels). In a DCE, a good or service is described with a number of relevant attributes and changing combinations of attribute levels. Respondents are asked to value situations against each other by choosing between several choice alternatives (i.e., choice sets). It is assumed that respondents take into account all information provided and select the option that has the highest value or utility for them. Thus from these selections, preferences are revealed indirectly through the respondents’ choices, making the relative importance of attributes and trade-offs derivable. Therefore, DCE studies show which attributes are driving individual’s preferences, which trade-offs respondents are willing to accept and how changes in attributes and attribute levels affect the respective preferences [15, 16].

### Selection of Attributes and Attribute Levels

The selection of attributes and attribute levels should adequately describe German re-opening strategies from the lockdown. As DCEs contain only a limited number of attributes (mostly 4–8), selection of attributes and attribute levels is a highly sensitive task [17]. We based our selection on existing Corona policies, ongoing public discussions and issues that had turned out to be relevant to the public as known from previous surveys [11]. We listed all relevant public areas locked down at the end of April (like retail trade, schools or sports facilities), potential protection measures (like social distancing, face masks or tracing app), and several health, economic and psycho-social outcomes. The final design of the DCE addresses [a] a health related attribute (ICU overload), [b] an economic attribute (gradient of unemployment rates), and [c] possible measures of re-opening strategies as well as protection measures supporting re-opening strategies (duration of school closure, re-opening bars and restaurants, quarantining elderly, and implementing a tracing app). The selection was done in several discussion rounds by a multi-disciplinary expert group consisting of health economists, epidemiologists, social scientists, psychologists and public health experts. To optimise the choice tasks, we pre-tested the DCE for face and theoretical validity in a health economic research group at Hannover Medical School, and both at University of Erfurt as well as the Federal Centre for Health Education resulting in the adaption of both attribute levels and descriptions. The final attributes and levels are shown in Table 1.

**TABLE 1:** Attributes and attribute levels included in the discrete choice experiment.

Attribute	Attribute levels		
Re-opening schools	Immediately	In 4 weeks	In 8 weeks
Re-opening restaurants and bars	Immediately	In 4 weeks	In 8 weeks
Tracing app	Voluntary		Mandatory
Quarantine for persons above 70 years	No		Yes
Available ICU capacities	Sufficient		Temporarily overloaded
Unemployment rate	5%	10%	20%

### Experimental Design

**FIGURE 1:**
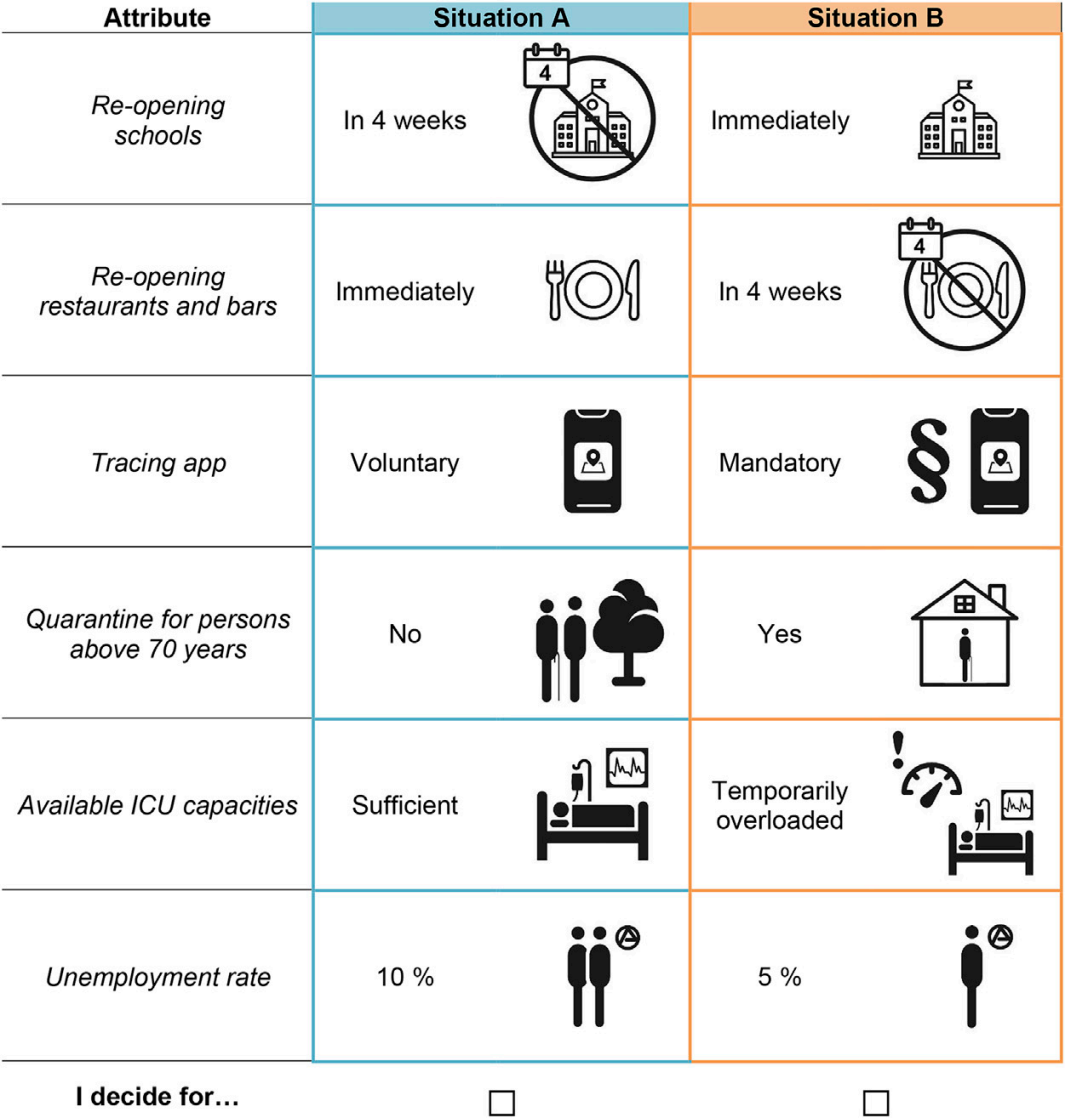
Example of a choice set.

The experimental design of a DCE refers to how the attributes and attribute levels are combined into choice alternatives and choice sets [15]. On the basis of 216 (i.e., 3^3^*2^3^) possible combinations of attribute levels a full factorial design confronting all respondents with all possible combinations of attribute levels is not feasible due to time frame and fatigue constraints. Therefore, we chose a fractional factorial design [15] with 16 choice sets comparing two alternatives each without opt-out. This reduces the number of choices used in the experiment while maximising the statistical efficiency (i.e., precision) of the design. Therefore, the 16 choice sets were randomly blocked into four questionnaire versions, each version containing four unlabelled choice tasks [18]. Blocking is an accepted statistical technique in DCE design to ensure balance among differing attribute levels [19]. Previous research suggests that respondents can efficiently handle approximately 10 choice sets at one time [20]. Versions of the survey were randomly allocated to respondents. We used SAS software (SAS Institute, Cary, NC, United States), which allows for the optimization of design efficiency, level balance, and choice task numbers. This design estimates a main effects model, while interaction effects are not estimable. See Figure 1 for an example of a choice set.

### Sampling and Data Collection

The DCE was integrated on April 28–29, 2020 into wave 9 of the COSMO survey [12]. The COSMO consortium conducts a weekly cross-sectional online-survey (15–20 min) on the COVID-19-situation for the general public. In each wave around 1,000 respondents complete an online questionnaire containing six parts: [a] socio-demographics and individual living situation, [b] knowledge about COVID-19, [c] risk perception, [d] protection behaviour, [e] trust in institutions and [f] attitudes towards containment policies. Wave 9 also included [g] the present study for preferences in exit strategies. An open panel maintained by the company Respondi (https://www.respondi.com/) was used to recruit a quota sample. The sample comprised German-speaking participants residing in Germany and is quota-representative of the German population regarding age (18–74 years), gender, and federal state [12]. Ethical approval for COSMO was obtained by University of Erfurt’s IRB (#202000302). Respondents were compensated for participation via Respondi at their usual rate. All individuals between 18 and 74 years of age completing the survey were eligible for inclusion into the analyses and comprise the final sample. All items and their instructions are detailed in the Supplementary Material.

The number of participants in the COSMO survey wave 9 exceeds the minimum sample size based on a rule-of-thumb calculation proposed by Johnson and Orme [21] and Orme [22]. Considering the number of choice tasks per respondent, the number of attributes and attribute levels, a sample size of 200 respondents was required.

### Data Analysis

The collected data for the DCE was imported into Stata version 15 (StataCorp LP, College Station, United States). First, descriptive statistics were conducted. Second, data to the DCE was analysed using conditional logit regression taking multiple choices of each respondent into account, which is a commonly used method for examining DCEs [15, 23]. However, as the conditional logit method has rather restrictive model assumptions, e.g. that it does not account for preference heterogeneity, we also used a latent class model (LCM). LCMs assume that there are respondents with similar choices and preferences, who can be grouped into latent classes. While preferences within a class are assumed to be homogeneous they differ between classes [24]. The number of latent classes is explorative, thus not initially determined, and is usually based on goodness-of-fit measures, such as log-likelihood ratio or information criteria as well as theoretical considerations [15, 25]. The calculation of relative importance of the attributes or attribute levels, respectively, allows for a comparison of preferences between classes. To characterise the identified latent classes, sociodemographic and attitudes items were analysed. Significant independent variables in the choice model point out that the attribute or attribute level has a significant impact on the preferences for exit strategies. Model results were expressed as parameter estimates (β) and their 95% confidence intervals (CI) as well as *p* values.

Additionally, we calculated marginal rates of substitution (MRS), so called trade-offs between two of the included attributes. The MRS can be calculated by partially differentiating the indirect utility function V regarding attributes $X_{i}$ and $X_{j}$ (from the included six attributes) and calculating their ratio [16, 26]:

$${\text{MRS}}_{X_{i}X_{j}}=\frac{\partial V/\partial X_{j}}{\partial V/\partial X_{i}}$$

We calculated the MRS of all attributes compared to the unemployment rate (attribute 6), as it is the only continuous outcome variable in the present DCE. Thus, e.g., the MRS of attributes 3 (tracing app) and 6 can be interpreted as the respondents’ willingness to accept an additional increase in the unemployment rate to avoid a mandatory tracing app (with the metric attribute “unemployment rate” being the denominator of this equation).

## Results

### Sample Characteristics

Table 2 shows the main characteristics of the respondents from the COSMO survey wave 9. 8, 933 participants were contacted by respondi, of whom 1,020 respondents participated in this survey [12]. As all respondents answered 4 choice sets, 4,080 choice sets were available for analysis.

**TABLE 2:** Characteristics of respondents (COSMO wave 9).

Characteristics		n (%)
Gender	Male	488 (47.8)
	Female	532 (52.2)
Age groups (years)	18–29	211 (20.7)
	30–49	357 (35.0)
	50–64	290 (28.4)
	65–74	162 (15.9)
Education	9th grade	112 (11.0)
	10th grade	353 (34.6)
	A levels	555 (54.4)
Households with children	No children	741 (67.6)
	Children	330 (32.4)
Migration background^a^	Yes	382 (32.2)
	No	692 (67.8)
Chronic diseases	Yes	359 (35.2)
	No	633 (62.1)
	I don’t know	28 (2.8)
Region	East	211 (20.7)
	South	281 (27.6)
	North-West	528 (51.8)

^a^ Migration background is defined as a combination of two variables: Respondents have a migration background in this study if either a) the language spoken at home is not German or b) either the respondents or at least one of their parents were not born in Germany.

### Preferences Derived from the Discrete Choice Experiment

**FIGURE 2:**
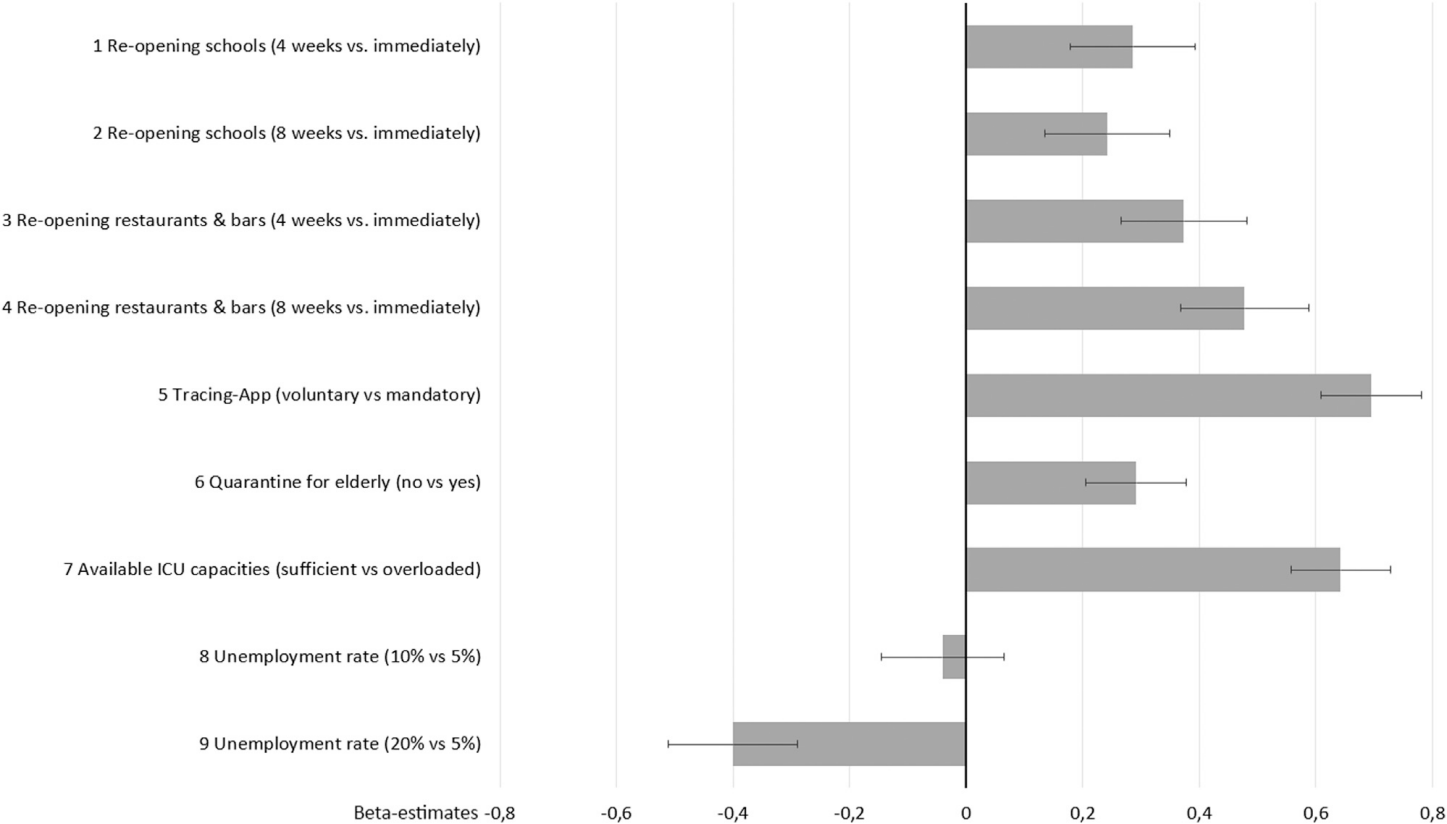
Preferences for exit strategies (conditional logit estimates and 95% confidence intervals).

Figure 2 presents the results of the conditional logit model. All attributes proved to be significant in all levels with the exception of 10% vs. 5% unemployment rate indicating that all attributes contributed to the respondents’ choices.

The most important attribute for the population is to avoid a mandatory tracing app, followed by the provision of sufficient ICU capacities which proves to be similarly important. Both attributes dominate all others. According to the respondents, gastronomy should be later re-opened than schools. Preventing a long-term 20% unemployment rate and avoiding the isolation of 70+ persons are relevant though utilities are lower than for most other attributes.

**FIGURE 3:**
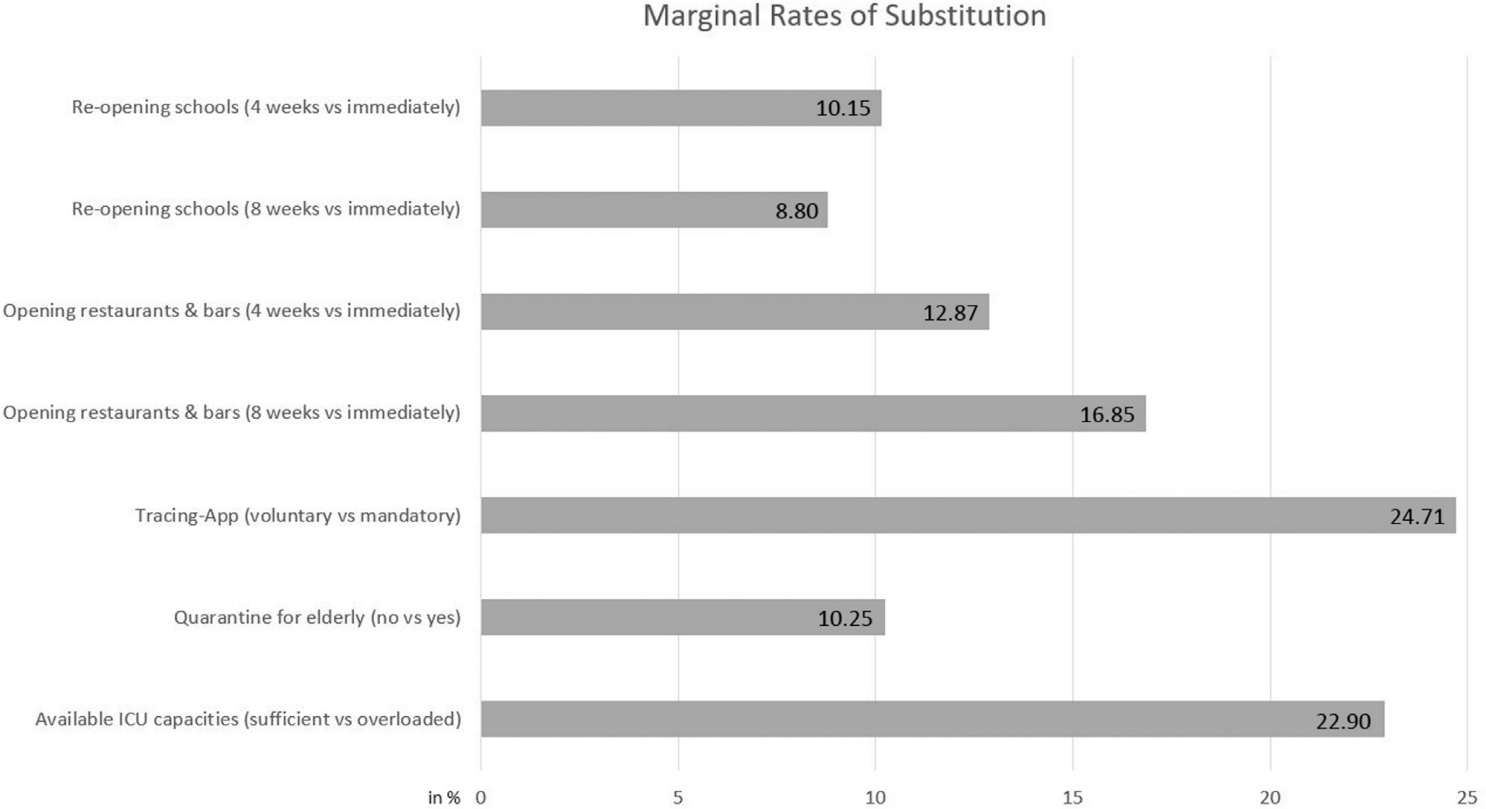
Marginal rates of substitution (compared to unemployment rate in %).

These findings are underscored by MRS indicating indifference in utility between the unemployment rate and the other attributes. As shown in Figure 3, respondents accept higher unemployment rates (than the current rate of 5%) if mandatory tracing app is prevented (25%), if ICU overload will be avoided (23%), or if isolating elder persons is avoided (10%).

### Subgroup Analyses

Subgroup analyses considering socio-demographic parameters show different choice patterns (see Supplementary Material). Concerning gender, avoiding high unemployment rates is more important to males than to females. Opposite, avoiding isolation of elderly persons is more important to females than to males. Comparing the age groups under 50 vs. over 65 years, the differences are most striking in preferences concerning the isolation of elderly persons. To persons aged 65+, avoiding isolation of elderly persons is by far the most important attribute, while in the younger age groups preferences against isolation nearly disappear. In contrast, preferences against high unemployment rates, overloading ICU capacities and a mandatory tracing app are clearly stronger in the age group under 50 than in the age group 65+. Respondents with vs. without children aged 6–13 years differ in their attitudes towards re-opening schools and/or gastronomy. While respondents with school children tend to be indifferent between early or late re-opening of schools, persons without school children have a clear preference for postponing school re-opening.

### Latent Class Model: Preference Heterogeneities

We identified two latent classes (presented in Table 3). Table 4 depicts members’ characteristics of the two classes.

**TABLE 3:** Estimation of the latent class model.

		Class 1		Class 2	
Class share		0.458		0.542	
Attributes		Coeff.	95% CI	Coeff.	95% CI
Re-opening schools (ref: immediately)	In 4 weeks	−1.261*	−2.388 – −0.134	0.503***	0.340 – 0.666
	In 8 weeks	−1.905**	−3.125 – −0.686	0.566***	0.421 – 0.711
Re-opening restaurants and bars (ref: immediately)	In 4 weeks	−1.768**	−2.920 – −0.617	0.616***	0.456 – 0.777
	In 8 weeks	−3.102***	−4.818 – −1.385	0.950***	0.762 – 1.137
Tracing app (ref: voluntary)	Mandatory	−1.814***	−2.875 – −0.752	−0.303***	−0.411 – −0.194
Quarantine for persons above 70 years (ref: no)	Yes	−0.477**	−0.777 – −0.176	−0.207***	−0.309 – −0.106
ICU capacities (ref: sufficient)	Temporarily overloaded	0.086	−0.326 – 0.497	−0.409***	−0.506 – −0.312
Unemployment rate (ref: 5%)	10%	−1.656***	−2.582 – −0.729	0.045	−0.081 – 0.172
	20%	−1.590***	−2.294 – −0.885	−0.085	−0.232 – 0.062
No. of observations	8,160				
No. of persons	1,020				
Log-likelihood	−2546.4383				
BIC	5098.543				
AIC	5098.543				

Notes: CI = confidence interval, Coeff. = coefficient; ICU = intensive care unit; BIC = Bayesian Information Criterion; AIC = Akaike Information Criterion; Significance: *p < .05, **p < .01, ***p < .001.

**TABLE 4:** Members’ characteristics in the two latent classes.

Characteristics		Class 1	Class 2
		*n* (%)	*n* (%)
Gender	Male	222 (47.5)	266 (48.1)
	Female	245 (52.5)	287 (51.9)
Age groups (years)	18–29	100 (21.4)	111 (20.1)
	30–49	160 (34.3)	197 (35.6)
	50–64	131 (28.1)	159 (28.8)
	65–74	76 (16.3)	86 (15.6)
Education*	9th grade	52 (11.1)	60 (10.9)
	10th grade	154 (33.0)	199 (36.0)
	A levels	261 (55.9)	294 (53.2)
Households with children	No children	341 (73.0)	400 (72.3)
	Children	126 (27.0)	153 (27.7)
Migration background***	Yes	164 (35.1)	164 (29.7)
	No	303 (64.9)	389 (70.3)
Chronic diseases***	Yes	153 (32.8)	206 (37.3)
	No	298 (63.8)	335 (60.6)
	I don’t know	16 (3.4)	12 (2.2)
Region	East	97 (20.8)	114 (20.6)
	South	134 (28.7)	147 (26.6)
	North-West	236 (50.5)	292 (52.8)
Risk perception***	Low	107 (22.9)	63 (11.4)
	Medium	317 (67.9)	414 (74.9)
	High	43 (9.2)	76 (13.7)
Frequency of contacts outside the own household per week***	0–1	217 (46.5)	292 (52.8)
	2+	250 (53.3)	261 (47.2)
Trust in institutions***	Low	111 (23.8)	113 (20.4)
	Medium	305 (65.3)	349 (63.1)
	High	51 (10.9)	91 (16.5)
Approval of lockdown policies***	Low	92 (19.7)	30 (5.4)
	Medium	362 (77.5)	496 (89.7)
	High	13 (2.8)	27 (4.9)

Notes: Significance: *p < .05, **p < .01, ***p < .001.

The shares of classes 1 and 2 are 0.458 and 0.542, respectively. The two classes differ clearly in their preferences. Class 1 has strong preferences for a rapid re-opening of schools and gastronomy, while rejecting mandatory tracing app and isolation of elderly persons. In contrast, class 2 members choose keeping schools and gastronomy closed for a longer period. They also have preferences against mandatory tracing app and isolation of elderly persons, but less than class 1 members. Avoiding long-term unemployment is highly relevant to class 1, while they do not mind overloading of ICU capacities. Preferences of class 2 are just reversed (compared to class 1): providing sufficient ICU capacities is quite important, while unemployment rate is not considered.

Classes 1 and 2 do not differ in most socio-demographic characteristics: Percentages of gender, age groups, region, and parenthood are quite similar in both classes. In contrast, we find differences between classes 1 and 2 in migration background and chronic disease (Table 4) as well as their attitudes, risk perception and behaviour: Class 1 members (a) have less trust in public institutions, media and science than class 2 members, (b) more often reject (actual or hypothetic) restrictions to liberties aiming at containing the pandemic, (c) have a lower risk perception (with risk defined as a combination of subjective probability to adopt SARS-CoV-2 and expected severity of COVID-19 disease if actually infected), and (d) have more social contacts with persons others than own household members.

## Discussion

Aim of the present study was to analyse the German population’s preferences for exit measures out of the lockdown in late April 2020. While there are several studies presenting likert scale or rating scale data [27–32], to our knowledge, this is the first (peer-reviewed) DCE addressing COVID-19 exit strategies (next to Jonker et al. [33] who are focusing on features of tracing apps). Our DCE combined strategies for re-opening public areas (schools and gastronomy), protection measures supporting re-opening strategies (tracing app and isolation of elder persons), and potential health (ICU capacities) as well as economic (unemployment) outcomes. In the following, trade-offs between exit strategies and outcomes are discussed, as DCEs (unlike rating scales) allow for identification and quantification of trade-offs supporting decision-making in dilemma situations.

### Exit Strategies

The respondents in general had no preference for an immediate re-opening of public areas. Moreover, from the latent class model we find two classes with distinct preferences. While the larger class 2 prefers late re-opening of public areas, class 1 (characterised by less trust in public institutions, less approval with restrictions to liberties and lower risk perception) clearly prefers early re-opening. Nevertheless, the results for the overall population correspond with direct preferences ratings, which were also obtained in COSMO wave 9: Immediate opening of retail trade (one of 15 public areas addressed in the COSMO questionnaire) was rated as most urgent, while education or sports facilities might be re-opened later, and cultural institutions might be locked down even longer [12]. Reluctance of the population to re-open public areas is also reflecting the scientific discourse in late April 2020 [34–36].

Protection measures included in the DCE were dismissed. This is in particular true for the adoption of a mandatory tracing app, but holds also for isolation of elderly persons. In fact, avoiding a mandatory app was the most important attribute for the population. Respondents would even accept a temporary overload of ICU capacities to avoid mandatory tracing. This corresponds with findings from COSMO wave 9, showing less than 50% of the respondents would be willing or at least consider to adopt a (voluntary) tracing app (while 22% would definitely not). The tracing app technology was not yet available in Germany at the end of April 2020.

Resulting from the public discussion, the German government reassured that no mandatory tracing app would be introduced [37]. Success of app-based tracing depends on high acceptance by the population; experts estimate necessary 60–80% participation rate of the population [38]. The examples of Austria and Singapore, where under 20% of the population have downloaded tracing apps [39], show both the importance and difficulties for public communication to increase acceptance. Trust in data protection measures and proof of tracing apps’ effectiveness might be crucial.

Isolation of persons aged 70+ is dismissed by the overall population. But subgroup analyses show clear differences between age groups. Preferences against isolation nearly disappear in persons under 50 years, while avoiding isolation is by far the most important attribute to respondents aged 65+. Though intended to protect the elderly population, isolation might be more perceived as restriction of (own) personal freedom.

### Health and Economic Outcomes

In Germany, there were sufficient ICU capacities due to generally high capacities in international comparison, and delaying elective surgeries as well as building extensive reserve capacities [40]. Also, Germany recorded less numbers of severe COVID-19 cases than several Western and Southern European countries [41, 42]. Moreover, infection numbers decreased since mid-March. Nevertheless, there were also fears of overload in the beginning, when infection numbers increased exponentially, and there were fears of large future infection outbreaks or even a second infection wave at the end of April [12]. Experiences of high death numbers and ICU overload from Italy, France and Spain [40, 41] might have influenced the respondents’ preferences.

Concerning the economic outcome, respondents tolerate a limited increase of the unemployment rate, but have clear preferences against an increase up to 20%. Several subgroups have higher than average preferences against unemployment, inter alia males (compared to females) and younger (compared to older) persons. Nevertheless, the utility from the health outcome surpasses the utility from the economic attribute for all subgroups (and thus for the overall population). However, the LCM indicates a substantial part of the respondents (i.e. class 1 members) preferring the economic outcome (low unemployment rate) over the health outcome (sufficient ICU capacities). Thus, attitude, risk perception and behaviour explain deviations from the overall population’s preferences.

### Trade-Offs Between Exit Strategies, Health and Economic Outcomes

Exit strategies affect both, health and economic outcomes. To understand population’s preferences, the trade-off between health and economics is crucial. In our experiment, the health outcome is more important to respondents than the economic outcome. Thus, respondents would rather accept a 20% unemployment rate for the next two years than an overload of ICU capacities at times. The point of indifference or maximum-acceptable unemployment is reached at an unemployment rate of 23%. Thus, at the end of April, respondents would only accept economy-boosting measures that risk ICU overload at times, if the expected long-term unemployment rate was more than 23% (which implies an extremely severe economic recession).

As a striking result of our DCE, we identified a) trade-offs between re-opening of public areas and the economic outcome on the one hand, and b) trade-offs between supporting measures and the health outcome on the other hand. a) Concerning lockdown measures effective in late April 2020, respondents prefer to postpone re-opening public areas for several weeks or months (intending to reduce infection risk and ICU utilization). The maximum-acceptable unemployment rate would be 17%, if gastronomy re-opening was postponed by eight weeks. An obvious interpretation of respondents’ preferences is that respondents see lockdown measures as a means to curb infection risk and ICU utilization. Thus, the trade-off between re-opening of public areas and the economic outcome might mirror the trade-off between health and economic outcome. b) While preferences towards re-opening of public areas support health outcomes (intending to reduce infection risk and ICU utilization by delaying re-opening), there are conflicting aims towards refusing the adoption of severe protection measures and improving health outcome. Respondents would even accept a temporary overload of ICU capacities to avoid mandatory tracing. Similarly, respondents have strong preferences against isolation of persons aged 70+, though they would accept quarantine measures if it helped to avoid ICU overload.

Finally, our DCE identified that there is no real trade-off between re-opening of public areas and adoption of additional protection measures. At the end of April 2020, the respondents prefer keeping public areas closed for several weeks or months and are at least sceptical about the adoption of severe protection measures.

### Limitations and Strengths

This DCE has some limitations. A formal process comprising systematic reviews and qualitative analyses such as expert interviews or focus group discussions, which is a regular part of designing a DCE [43, 44], was not feasible: The COVID-19 pandemic is an unprecedented experience and characterized by rapid changes in the dynamic progression, knowledge about the virus and infection routes and evidence of effective protection measures. The selection of attributes and attribute levels was done in several discussion rounds by a multi-disciplinary expert group. The final version was checked in extensive pre-tests. To guarantee fast recruitment, respondi was commissioned to recruit for and facilitate our survey via their open panel. The sample is stratified for the variables sex, age and federal state, while other variables such as income or education were not taken into account.

On the other hand, this study has some strength. As the DCE is embedded in the COSMO survey wave 9, it is ensured that respondents are quota-representative for the German population. Furthermore, this survey was conducted within only two days (April, 28–29), which is crucial to gain comparable results in a dynamic setting. Although the DCE faces some limitations, it provides valuable insights into the population’s preferences for or against re-opening public areas.

## Conclusion

The DCE delivers crucial results for the overall population’s preferences, but also for different preferences in subgroups.

(1) Comparing exit strategies, we find no real trade-off between re-opening of public areas and adoption of additional protection measures. The population prefers cautious re-opening strategies and is at least sceptical about the adoption of severe protection measures.
(2) Utility from health outcome is higher than from economic outcome though there is a substantial subgroup (latent class 1) with reversed preferences.
(3) We identified subgroups with strikingly different utility from specific attributes affecting them more than the rest of the population. Government should pay attention to the preferences of subgroups and balance interests between subgroups and the rest of the population. Moreover, persons with less trust in public institutions and low risk perception might be challenging for future public COVID-19 policies. Knowing population’s preferences can be helpful for decision-making and for balancing of interests. It can serve to insert measures into the general national situation and thus to build understanding and trust.
(4) To map preference changes caused by alterations of the COVID-19 pandemic further DCEs will be conducted in subsequent COSMO waves.

## Data Availability

The raw data supporting the conclusions of this article will be made available by the authors, upon reasonable request.
