# Supplementary material for: Public Preferences for Exit Strategies From COVID-19 Lockdown in Germany—A Discrete Choice Experiment
Source: Int J Public Health. 2021 Mar 19;66:591027. doi: 10.3389/ijph.2021.591027 (PMC8565260; doi:10.3389/ijph.2021.591027)

International Journal of Public Health

**Public preferences for exit strategies from COVID-19 lockdown in Germany – a discrete choice experiment**

**Additional measures**

Participants gave sociodemographic information regarding age, gender, federal state of residence, education, number of children, and chronical illness status. Participants further indicated whether a language other than German is the primary language in the household and whether the participant or his or her parents were born outside Germany. Participants indicated how often they met other individuals which did not belong to their household in the last week on a 4-point scale ranging from 1 = “not at all”, 2 = “once a week”, 3 = “two to four times per week”, and 4 = “at least five times per week”. Subjective risk regarding COVID-19 was assessed with two items: “How would you rate an infection with the novel coronavirus for yourself?”, 7-point scale with 1 = “completely harmless” to 7 = “extremely harmful”. “How likely do you think you will be infected with the novel coronavirus?”, 7-point scale with 1 = “extremely unlikely” to 7 = “extremely likely”. The participants also answered a question regarding their perceived Corona preparedness on a 7 - point scale (“How certain or uncertain do you feel about which protective measures are suitable to prevent infection with the new coronavirus?”, 1 = “very uncertain” to 7 = “very certain”). Trust in institutions (e.g. governmental institutions and the health system) was measured on a 7-point scales ranging from 1 = “very little trust” to 7 = “very much trust”. Policy agreement was rated on a 7-point scale, ranging from 1 = “strongly disagree” to 7 = “strongly agree” ( e.g., “The government should restrict personal liberties to fight the novel coronavirus.”). All items and their instructions are available online from the Open Science Framework (https://osf.io/3bsf9/?view_only=a47917c478d24137be6cd0ce9ddbec53).

**Fig S1a** Preferences by gender (conditional logit estimates and 95 % confidence intervals)


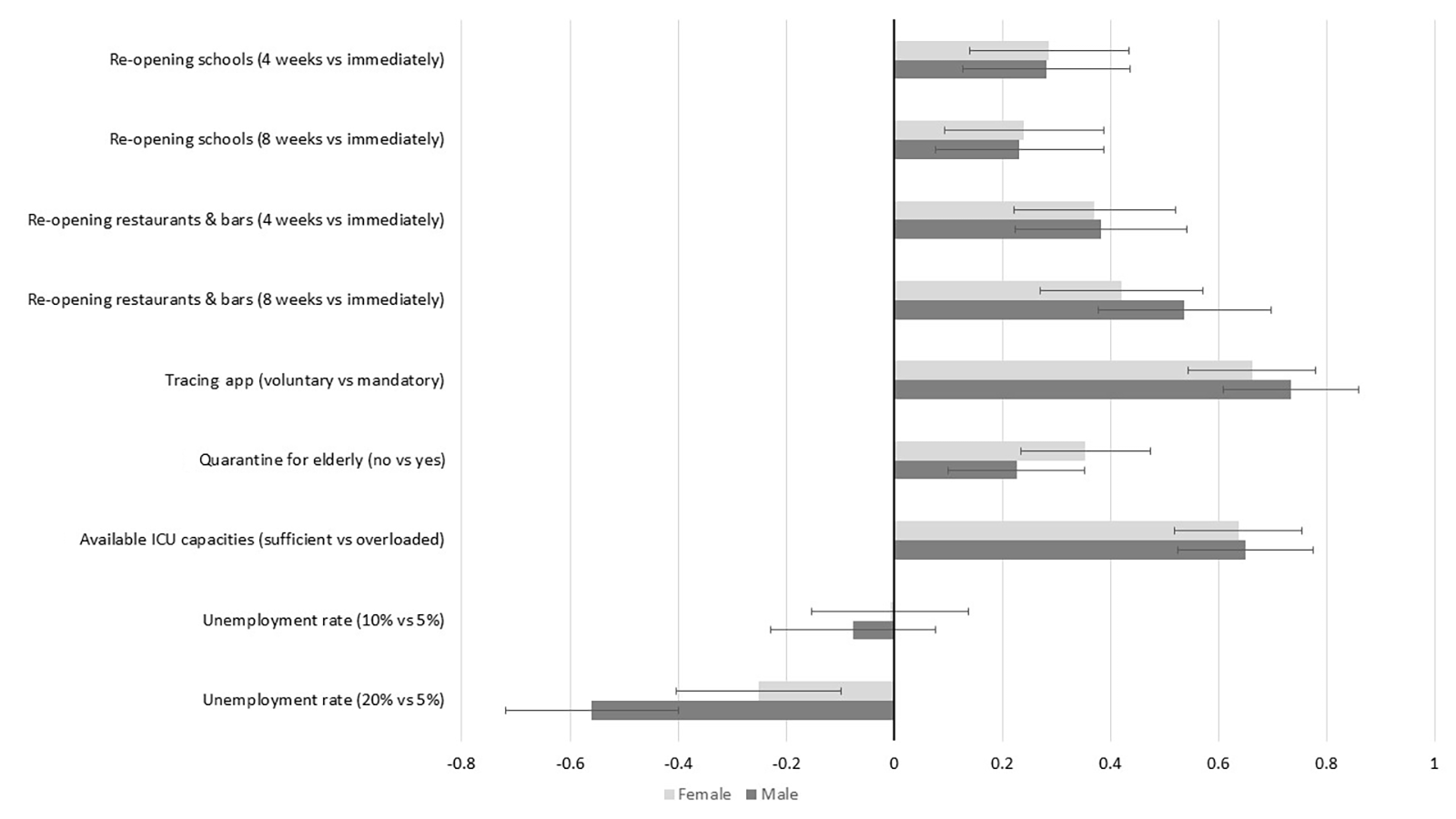


**Fig S1b** Preferences by age groups (conditional logit estimates and 95 % confidence intervals)


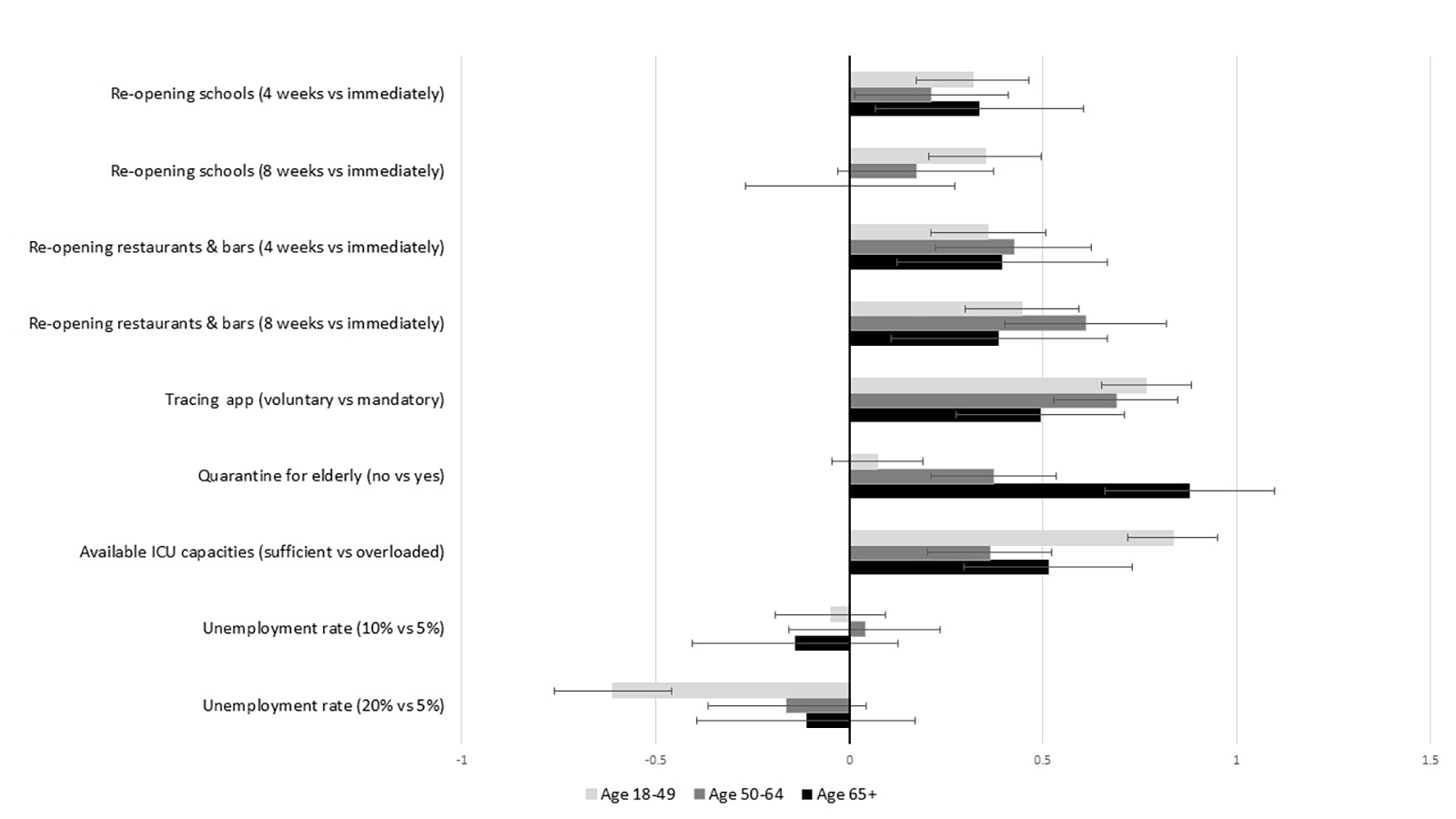


**Fig S1c** Preferences by household status with and without children (conditional logit estimates and 95 % confidence intervals)


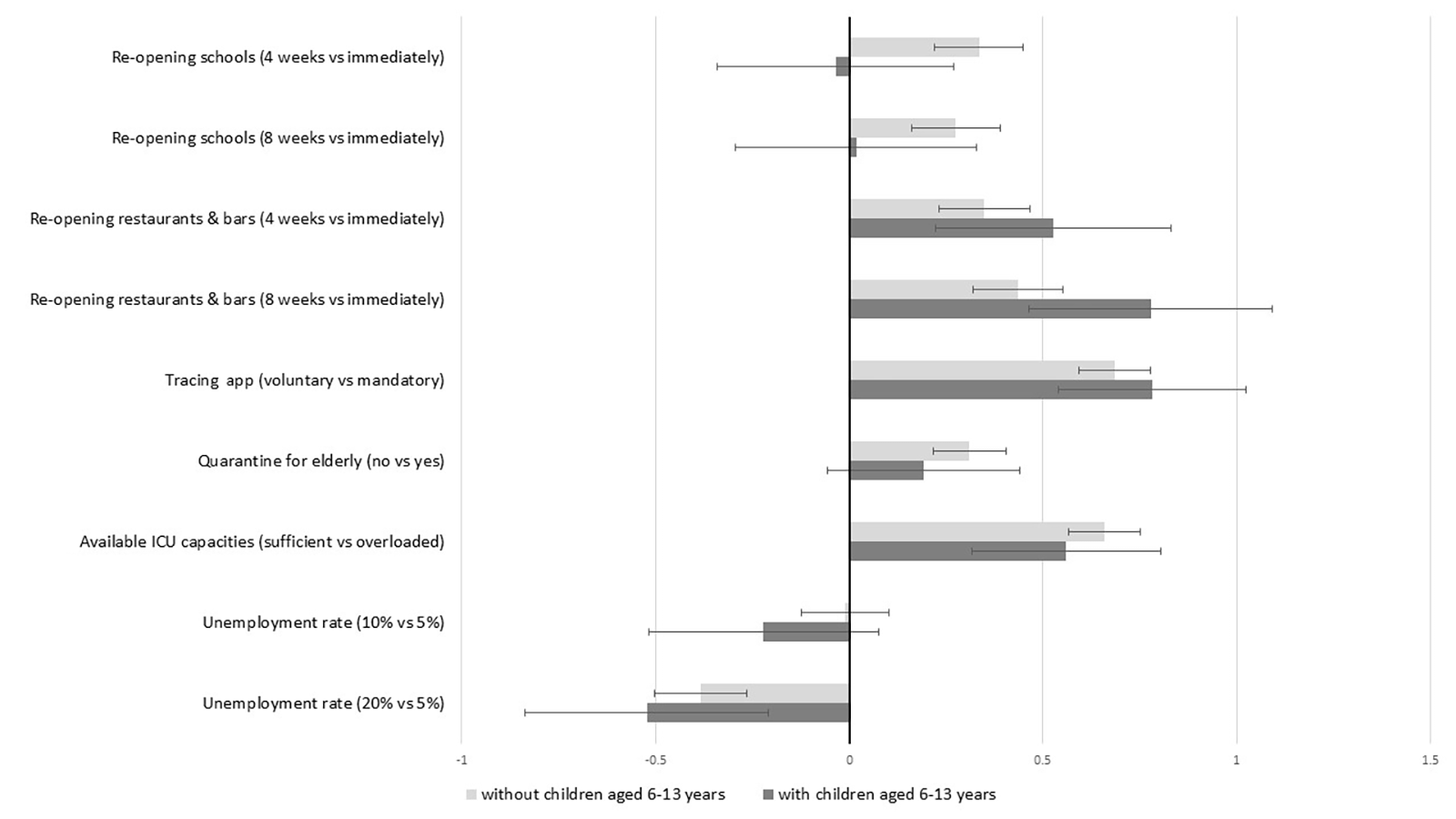

Supplement: Supplementary file 1 [file Table1.docx]
